# Neutralization of Industrial Water by Electrodialysis

**DOI:** 10.3390/membranes11020101

**Published:** 2021-01-31

**Authors:** Oleksandr Petrov, Natalia Iwaszczuk, Tina Kharebava, Irina Bejanidze, Volodymyr Pohrebennyk, Nunu Nakashidze, Anton Petrov

**Affiliations:** 1 Faculty of Management, AGH University of Science and Technology, 30-059 Kraków, Poland; niwaszcz@zarz.agh.edu.pl; 2 Department of Chemistry, Batumi Shota Rustaveli State University, Batumi, GE 6010, Georgia; tina.kharebava@bsu.edu.ge (T.K.); irina.bejanidze@bsu.edu.ge (I.B.); 3 Department of Ecological Safety and Nature Protection Activity, Lviv Polytechnic National University, 79013 Lviv, Ukraine; volodymyr.d.pohrebennyk@lpnu.ua; 4 Department of Agroecology and Forestry, Batumi Shota Rustaveli State University, Batumi, GE 6010, Georgia; nunu.nakashidze@bsu.edu.ge; 5 Department of Information Systems, Kuban State Agrarian University named after I.T. Trubilin, 350044 Krasnodar, Russia; petrov.a@kubsau.ru

**Keywords:** electrodialysis, bipolar membrane, solution pH correction, water dissociation

## Abstract

The process of non-reagent adjustment of the pH of a NaCl solution (0.5 g/L) of different acidity was investigated by the method of bipolar electrodialysis on a device operating according to the K-system (concentration). The experiments were carried out in the range pH = 2.0–12.0 with monopolar cation-exchange MK-40 (for alkaline solutions) or anion-exchange MA-40 (for acidic solutions) and bipolar MB-2 membranes. The regularities of the change in the pH of the solution on the current density, process productivity and energy consumption for the neutralization process have been investigated. Revealed: with different productivity of the apparatus (Q = 0.5–1.5 m^3/^h), in the range of pH 3.0–11.0, with an increase in the current density, a neutral pH value is achieved. It has been shown that at pH above 11.0 and below 3.0, even at high current densities (i > 20 A/m^2^), its value cannot be changed. This is due to the neutralization of the H^+^ or OH^−^ ions generated by the bipolar membrane by water ions, which are formed as a result of the dissociation of water molecules at the border of the monopolar membrane and the solution under conditions when the value of current exceeds the limiting value.

## 1. Introduction

Environmental protection and rational use of natural resources are becoming increasingly important today to prevent pollution of water bodies with industrial wastewater. Wastewater containing mineral acids or alkalis is neutralized before being discharged into water bodies or before being used in technological processes. Neutralization is carried out in order to prevent corrosion of materials of sewage treatment facilities, disruption of biochemical processes in biological oxidants and in water bodies, as well as for precipitation of heavy metal salts from wastewater [1,2,3].

According to the degree of aggressiveness, industrial wastewater is divided into slightly aggressive (weakly acidic with pH = 6.0–6.5 and slightly alkaline with pH = 8.0–9.0), highly aggressive (strongly acidic with pH <6 and strongly alkaline with pH >9) and non-aggressive (pH = 6.5–8.0) [4,5]. When industrial wastewater is discharged into a reservoir or city sewer network, mixtures with pH = 6.5–8.5 are considered to be practically neutral. Considering the neutralizing ability of the reservoir and the alkaline reserve of urban wastewater, wastewater with a pH of less than 6.5 and more than 8.5 should be neutralized.

Neutralization can be carried out by various methods: mixing acidic and alkaline waste waters, adding reagents, filtering acidic waters through neutralizing materials, absorption of acid gases in alkaline waters, or absorption of ammonia in acidic waters [6,7]. Precipitation may form during neutralization. Various reagents are used in the chemical treatment of industrial wastewater. To neutralize acidic waters, the following can be used: NaOH, KOH, Na_2_CO_3_, ammonia water, CaCO3, and MgCO_3_, and for alkaline waters—various acids or acid gases, for example, waste gases containing CO_2_, SO_2_, NO_2_, N_2_O_3_, etc. [8,9,10,11].

In many industrial and agricultural processes (washing water treatment, industrial wastewater treatment, caustic soda preparation, chlorine production, whey processing, and water preparation for use in agriculture and water supply systems), liquid treatment systems need to be developed to regulate pH [12,13,14,15].

When adjusting the pH of liquids with chemical reagents [16] and when preparing water for clarification, acidity is controlled by dosing alkaline or acid solutions. In control systems for growing plants under cover, an automatic measuring device of alkali and acid solutions with a concentration of 20–30% is also proposed. Despite the variety of methods for dosing chemical reagents, their use, as a rule, leads to great inconvenience in work for the following reasons:

✓ the formation of a large amount of sediments as a result of changes in acidity;
✓ disruption of dosing mechanism operation due to clogging of holes;
✓ the need to prepare reagent solutions of a certain concentration;
✓ inaccuracy in predicting and obtaining acidity values.

In addition, increasing the scale of production consumption requires additional reagent costs and, accordingly, increases the cost of water treatment. In addition, waters with pH~7, in case of their increased salinity of large water volumes, pose an environmental threat [17,18,19].

Membrane wastewater treatment methods are an alternative to reagent pH control systems. Membrane technologies belong to the category of resource-saving technologies, the use of which makes it possible to improve the quality of discharged wastewater, reduce the quantitative discharge of pollutants into water bodies, and minimize the intake of natural water due to the possibility of reusing treated wastewater in closed water supply systems [20,21,22].

According to the forecasts of the development of the world economy, membrane technology is regarded as the technology of the future [23,24,25]. It has been widely used in many industries and agriculture. Membrane processes are used in almost all spheres of human activity, since they ensure high efficiency of processes, namely: reduce the cost of materials, raw materials and energy; increase the thermal and energy potential; provide the population with drinking water; protect the environment, etc. [26,27,28,29]. Electrodialysis is one of the methods of membrane technology for the separation and purification of liquids [30,31,32]. Electrodialysis is an environmentally friendly [33]. The application of this method allows you to successfully carry out the processes of desalination and concentration of solutions, obtaining drinking water from salt water, treatment of natural and industrial wastewaters, etc. It is widely introduced in the energy, electronic, chemical, and food industries, and in medicine, agriculture and other spheres of human activities [34,35]. The world market of membrane technologies already occupies a significant segment of the world economy. According to experts’ forecasts, the world demand for membranes will grow steadily. The annual volume of global sales of membranes and membrane equipment has recently increased by 10–12% and by the beginning of the XXI century amounted to over 11 billion US dollars.

The basis of electrodialysis is the selective transport of ions of a dissolved substance through ion-exchange membranes under the influence of an electric field. The driving force of this process is the gradient of the electric potential on both sides of the membrane. The scope of the electrodialysis method expanded with the use of bipolar membranes. A bipolar membrane is a two-layer composite membrane consisting of layers of cation- and anion-exchange membranes that are directly in contact with each other [36].

**Figure 1:**
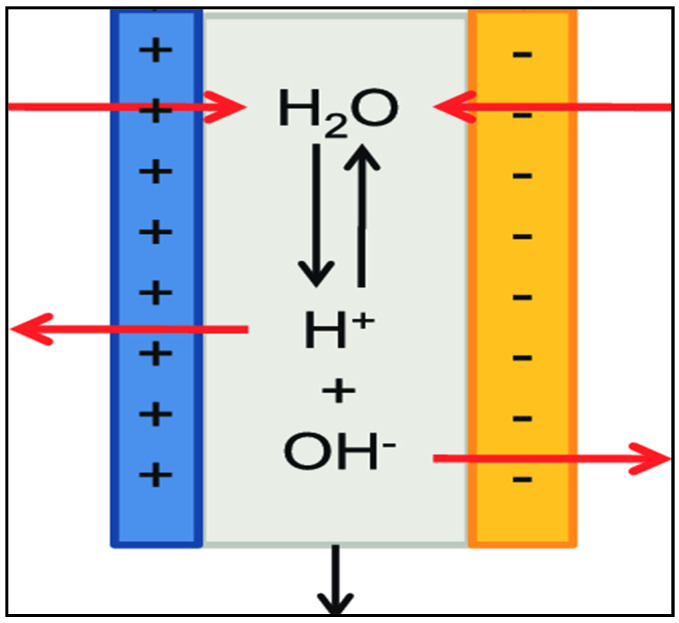
Generation of H^+^ and OH^-^ ions by the bipolar membrane.

**Figure 2:**
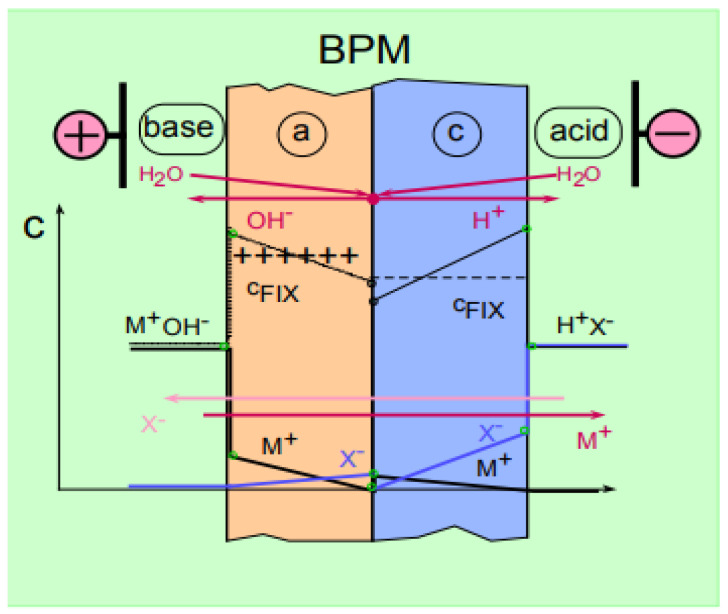
Schematic concentration profiles in a bipolar membrane.

When the bipolar membrane is located with the cation-exchange side to the cathode and the anion-exchange one to the anode, the membrane generates H^+^ and OH^-^ ions due to the splitting of water molecules, even with a small electric field voltage at the interface between the layers [36] (Figure 1 and Figure 2).

This ability of the bipolar membrane is successfully used to obtain acids and bases from salt solutions or to carry out other chemical transformations involving hydrogen and hydroxyl ions. The decomposition of water in such systems is a “working” process and the efficiency of the applied electromembrane technology depends on the overvoltage and current efficiency with which the H^+^ and OH^−^ ions are generated.

Currently, electrodialysis with bipolar membranes is used not only to obtain acids and bases, but also for reagent-free regulation of the pH of various liquids [35,36].

It is known that when growing plants on artificial substrates, it is necessary to regularly monitor the pH value. For most plants, the acidity of the medium is optimal, pH 5.5–6.5.

The authors of [37] successfully solved the problem of controlling the acidity of hydroponic solutions by the method of bipolar electrodialysis. This method, in contrast to the chemical method, allowed them to obtain stable indicators of the acidity of the solution without changing the organoleptic properties.

Many scientists and engineers have attempted to develop fluid treatment systems in industrial and agricultural processes to control pH (preparation of washing water, treatment of industrial effluents, processes for producing caustic soda and chlorine, treatment of milk whey, and preparation of water for use in agriculture and water supply systems).

Enterprises, consumers of large amounts of water, have certain requirements for water. For example, the efficiency of the coagulation process in water treatment systems is largely dependent on the pH of water under treatment, since only at the optimum pH = 10.0–10.5, the minimum solubility and maximum strength of the resulting hydroxides is achieved. Also, washing wastewater, obtained after acid-base regeneration of ion-exchangers and those used in thermal and nuclear energy, before being discharged into the sewer, requires adjusting the acidity to a neutral media [38].

However, it should be noted that for each specific case of using the electrodialysis process, depending on the goal, it is necessary to select the type of electrodialysis apparatus and membranes, the hydraulic circuit, and electrical parameters of the process [39,40,41,42].

Thus, the issue of adjusting the pH of saline solutions and industrial effluents, without the use of chemical reagents, is very relevant, since it will simplify the technological schemes in use, reduce the volume of effluents and the cost of chemical reagents, and also concentrating and returning valuable products to the technological cycle will make the process more economical and, accordingly, reduce the risk of environmental pollution.

The purpose of this work: to study the process of reagent-free pH adjustment (up to neutral) of saline solutions of various acidities in a multi-chamber pilot plant operating according to the K (concentration) system, using the bipolar electrodialysis method, in order to reduce the aggressiveness of industrial effluents before being discharged into the sewer or to return the process products to the technological cycle production

The novelty of the work: Most of the work on adjusting the pH of solutions was carried out on model installations of small volume, consisting of a maximum of five to seven working chambers, using bipolar and anion-exchange membranes. In this work, the pH adjustment of solutions was carried out by the method of electrodialysis with bi- and monopolar ion-exchange membranes, on an experimental setup with 50 working chambers operating according to the K-concentration system. Through this system, the working solution was not supplied to the concentration chambers; a solution was formed in these chambers due to the generation of OH ions and hydrated sodium ions by the bipolar membrane, transferred through the cation exchange membrane, that is, alkali is formed in the concentration chambers. When an anion-exchange membrane is used in the concentration chambers, acid is formed due to the generation of H^+^ ions and hydrated chlorine ions by the bipolar membrane. In parallel, the electroosmotic transfer of water to the concentration chambers takes place.

## 2. Methods and Materials

**Figure 3:**
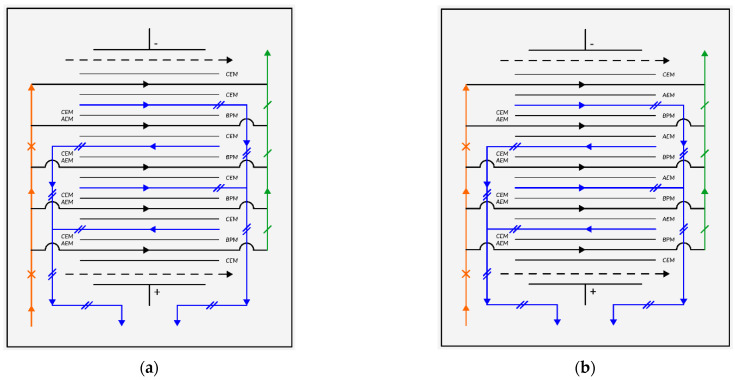
Schematic diagram of the electrodialysis process for the correction of acidic (**a**) and alkaline (**b**) solutions: AEM-anion, CEM-cation, and bipolar membrane (BPM); ----x---- initial solution, ---/-- product and ---//--- concentrate.

Investigation into the change in pH of salt solutions of different acidity was carried out on an experimental electrodialysis apparatus, consisting of two platinized titanium electrodes and a membrane package located between them which, when adjusting the pH of alkaline salt solutions, consisted of alternately arranged cation-exchange MK-40 and bipolar MB-2 membranes (Figure 3a), and in the case of acidic solutions—anion-exchange MA-40 and bipolar MB-2 membranes (Figure 3b).

**Figure 4:**
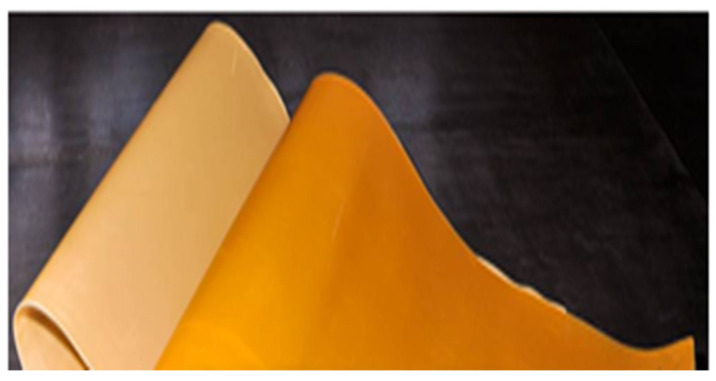
Cation-exchange MK-40.

**Figure 5:**
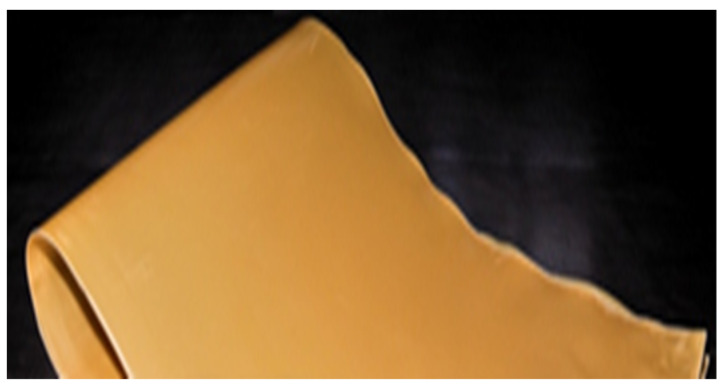
Bipolar MB-2 membrane anion-exchange membrane.

The membranes are manufactured at JSC Shchekinoazot (Shchekino, Russia). The MK-40 (Figure 4) cation-exchange membrane was made of the KU-2–8 strongly acidic cation exchanger composition containing sulfo groups and polyethylene; the MA-40 (Figure 5) anion-exchange membrane was made of the EDE-10 P anion exchanger composition containing quaternary ammonium bases (20%), secondary amines and polyethylene. The bipolar membrane of the MB-2 brand is made on the basis of KU-2 gel-type sulfocationionite and AB-1 gel-type benzyltrimethylammonium anionite containing the groups: -SO_3_H,–N(CH_3_)_2_.

**Figure 6:**
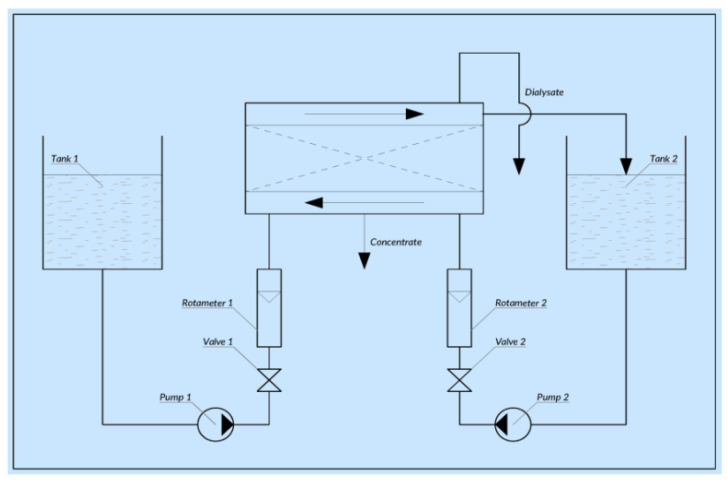
Scheme of the process for adjusting the pH of technological solutions.

The scheme of the process for adjusting the pH of technological solutions is shown in Figure 6.

The working solutions with different pH values were prepared by adding sodium chloride (0.5 g/L), sodium hydroxide or hydrochloric acid solutions to the solution. The pH of the initial solutions varied in the range of pH = 2.0–12.0.

In this work, the pH adjustment of solutions were carried out in an experimental setup with 50 working chambers operating according to the K-concentration system. Through this system, the working solution was not supplied to the concentration chambers; a solution was formed in these chambers due to the generation of OH^-^ ions and hydrated sodium ions by the bipolar membrane, transferred through the cation exchange membrane, that is, alkali is formed in the concentration chambers. When an anion-exchange membrane is used in the concentration chambers, acid is formed due to the generation of H^+^ ions and hydrated chlorine ions by the bipolar membrane. In parallel, the electroosmotic transfer of water to the concentration chambers takes place.

The thickness of each chamber is 1.2 mm, and the effective working area of one membrane is 0.124 m^2^. The membrane package was hydraulically assembled in parallel, which provides the same process conditions in all chambers. The initial solution was not supplied to the concentration chambers. The movement of fluid in these chambers was carried out due to the electroosmotic transfer of the solvent from the dialysate chamber.

The NaCl concentration was determined by the Cl^-^ ion recalculated to NaCl. The Cl^-^ ion content was determined by the argentometric method in the presence of the K_2_CrO_4_ indicator. The concentration of alkali and acid was determined by acid-base titration in the presence of the phenolphthalein indicator.

Experiments on adjusting the pH of the initial solutions of different acidity were carried out at a constant voltage on the apparatus and a constant flow rate of the initial solution into the dialysate chambers (flow rate Q = 0.5–1.5 m^3^/h). The process of electrodialysis correction of the acidity of each initial solution was carried out until the neutral medium of the solution was reached in the dialysate chamber, which was achieved with an increase in the voltage on the apparatus. The voltage value was controlled with a voltmeter, the current in the apparatus with an ammeter, and the consumption of the initial solution into the dialysate chambers with a rotameter.

For each hydraulic mode, by changing the value of the applied voltage to the apparatus, the optimal electrical parameters of the process were set and the following process indicators were calculated on their basis: operating capacity (Q) and energy consumption (W) per process. These indicators were calculated using the formulas:

1. Process performance:

$$Q=\frac{V\left(product\right)}{\tau \cdot S}l/\left(h\cdot m^{3}\right)$$

where:

✓ *V* (product)—product volume, l;
✓ *τ*—time, h;
✓ *S*—membrane area, m^2^

2. Energy consumption:

$$W=\frac{U\cdot I\cdot \tau }{V}\;\mathrm{kW}\cdot h/m^{3}$$

where:

✓ *I*—amperage, kW;
✓ *τ*—time, h;
✓ *V*—product volume, m^3^;
✓ *U*—voltage across the electrodialyzer

## 3. Results and Discussion

Reagentless acidity adjustment of saline solutions with initial pH = 2.0–12.0 was studied depending on the applied voltage to the apparatus. When alkaline salt solutions were passed through an electrodialysis apparatus, the pH of the solutions in the dialysate chambers decreased in accordance with the increase in voltage, almost to a neutral value. The alkaline working solutions are neutralized with hydrochloric acid formed in the dialysate tract by chlorine ions of the working solution and hydrogen ions migrated from the cationic layer of the bipolar membrane. At the same time, alkali is formed in the concentration chambers by sodium ions of the working solution and hydroxyl ions migrated from the anion layer of the bipolar membrane.

**Figure 7:**
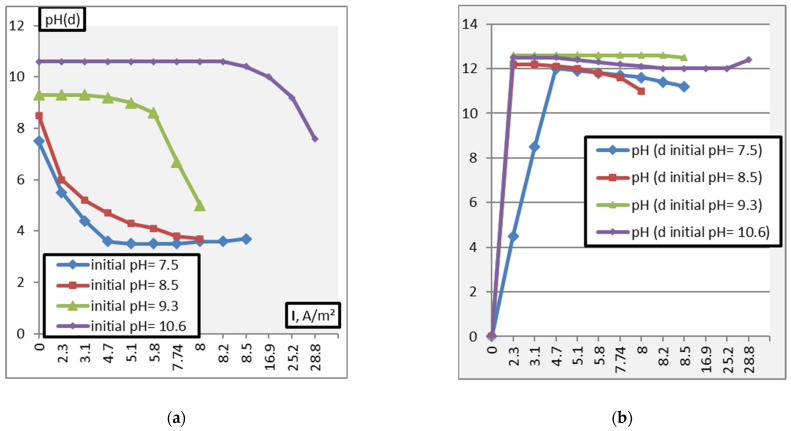
Dependence of the pH. (**a**)—pH(d), (**b**)—pH(c)) adjustment of alkaline solutions on the current density (d—dialysate, c—concentrate).

**Figure 8:**
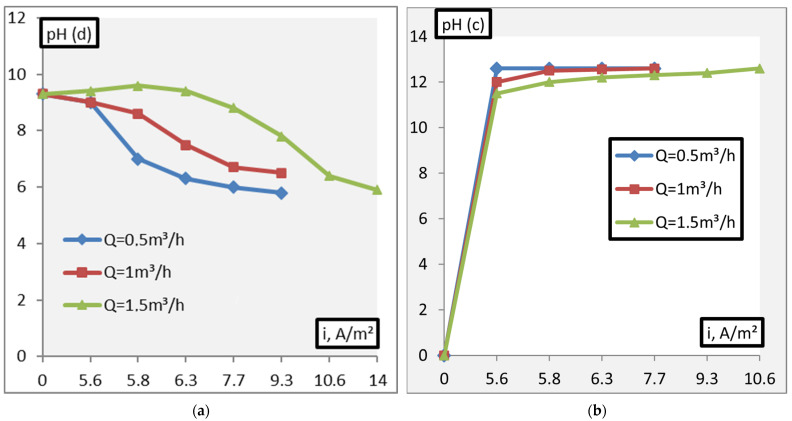
Dependence of the pH. ((**a**)—pH(d), (**b**)—pH(c)) adjustment of alkaline solutions on the current density and process performance (initial pH = 9.3)

**Figure 9:**
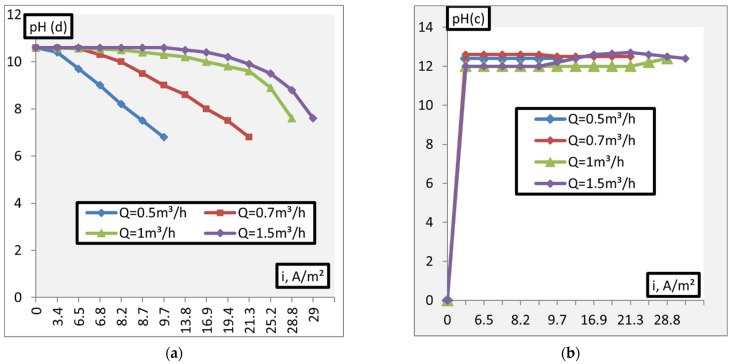
Dependence of the pH. ((**a**)—pH(d), (**b**)—pH(c)) adjustment of alkaline solutions on the current density and process performance (initial pH = 10.6).

**Figure 10:**
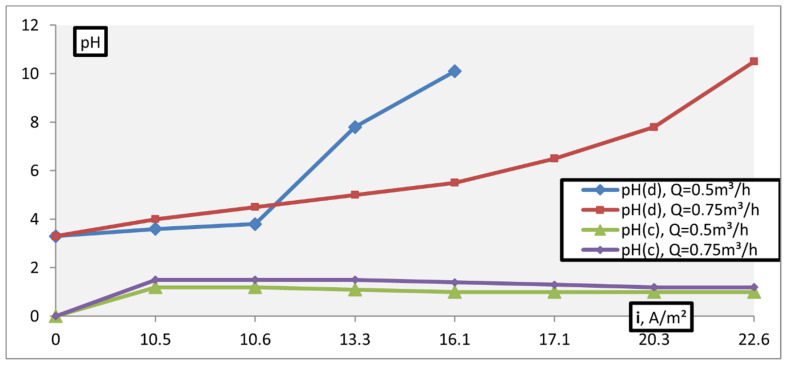
pH adjustment of acidic solutions (pH = 3.3).

**Figure 11:**
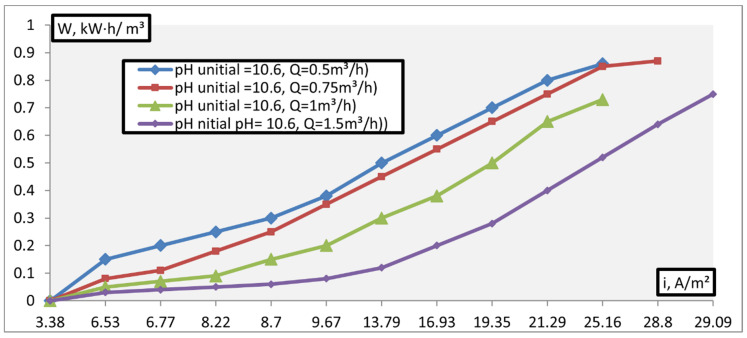
Dependence of the energy consumption of the process of adjusting acidic solutions on the current density.

The process of adjusting the pH of solutions was studied at different operating capacities (Q = 0.5–1.5 m^3^/h) of the apparatus. The results obtained during the experiment are shown in Figure 7, Figure 8, Figure 9, Figure 10 and Figure 11.

Figure 7 shows the dependence of the pH adjustment of alkaline solutions on the current density. The membrane package is assembled from MK-40 and MB-2.

The analysis of the study results showed that the neutralization of weakly alkaline solutions (pH = 7.5–8.5) can be achieved under conditions of current density values (i ≈ 1 A/m^2^). Further current density increase leads to a shift of pH in the solutions (dialysate) from a neutral (pH~7) to acidic environment. An increase in the alkalinity of the working solutions in the pH range of 8.5–11.0 requires a significant increase in the current density to neutralize these solutions: The greater the deviation of the pH value of the initial solution from neutral, the higher the voltage should be.

Figure 8 and Figure 9 show the results of pH adjusting of solutions with an initial value of pH = 9.3 and pH = 10.6, respectively, with different operating capacities of the apparatus. It was found that in conditions of constant productivity, an increase in current density increases the intensity of pH decreasing, and an increase in productivity increases the value of current density required to neutralize the solution. Besides, the intensity of lowering the pH of the solution is greater, the higher the current density; and it is less, the greater the productivity of the apparatus.

During electrodialysis of the initial solution with pH >11, a decrease in the pH of the product (dialysate) was practically not achieved. This is probably due to the operation of the installation under conditions above the limiting current value, when the splitting of water molecules occurs at the interface between the monopolar membrane and the solution.

Under the operating conditions of the installation up to the limiting current value when using only cation-exchange and bipolar membranes, acid is formed in the dialysate chamber due to H^+^ ions generated by the bipolar membrane and chlorine ions of the working solution, and the acidity of alkaline solutions decreases. Under extreme conditions, at the interface between the cation exchange membrane and the solution, water molecules are split into H^+^ and OH^−^ ions. H^+^ ions are transported by the cation exchange membrane, and the resulting OH- ions neutralize the H^+^ ions generated by the bipolar membrane. As a result of this process, the acidity of the working solution does not decrease [42].

During the experiment on alkaline salt solutions, in the concentration sections of the apparatus, in contrast to dialysate chambers, the formation of a 5–6% sodium hydroxide solution, with a volume of no more than 2% of the product volume, took place.

In addition to alkaline solutions, the possibility of adjusting the pH of acidic saline solutions was studied. To solve this problem, the electrodialysis apparatus was equipped with bipolar- and anion-exchange membranes.

Correction of the acidity of sodium chloride solutions with pH = 2.7 and pH = 3.3 was carried out. The initial solution was supplied only to the dialysate chambers of the apparatus.

In these chambers, when voltage is applied, sodium hydroxide is formed due to Na^+^ cations of the work solution and OH^-^ anions, generated by the bipolar membrane. As a result of the experiment, it was found that when processing a solution with pH = 2.7 even at a high current density (i >20 A/m^2^), the pH of the product does not change, which is probably due to the neutralization of hydroxyl ions generated by the bipolar membrane by hydrogen ions obtained by splitting water at the border of a monopolar membrane with a solution under extreme operating conditions of the device.

The results obtained during the electrodialysis treatment of an acidic initial (working) solution with pH >3.3 are shown in Figure 10.

As follows from the data obtained, the intensity of change in the acidity of the solution depends on the current density and apparatus process performance: With an increase in the value of these indicators, the pH of the product increases and, consequently, the energy consumption of the process increases. Figure 11 presents data on dependence of the energy consumption of the process of adjusting acidic solutions on the current density.

## 4. Conclusions

The reagent-free process of pH correction of NaCl solutions of different acidities was carried out by the method of bipolar electrodialysis.

It was found that in the range 3.0 ≤ pH ≤ 11.0, the change in the acidity of the working solution to a neutral value depends on the initial pH value, current density and the performance of the apparatus.

It was found that the greater the deviation of the pH of the initial solution from the neutral value and the higher the productivity of the installation, the higher the current density and energy consumption for the neutralization process should be. It has been established that when the apparatus is operating in the overcurrent mode, bipolar electrodialysis is ineffective for correcting the pH of solutions, since the H^+^ and OH^−^ ions generated by the bipolar membrane are neutralized by the corresponding ions that are formed at the interface between the monopolar membrane and the solution during water dissociation. The data obtained in this work can be used to simulate the processes of pH correction in solutions. The proposed technology for pH correction can find application in the regeneration of water used to feed steam circuits of steam boilers, in the processing of natural water, juice and wine, or in the production of ultrapure water. etc. The development of reagent-free methods for the acidification and decarbonization of natural waters and carbonate-containing technological solutions is a very urgent task. Its solution would make it possible to harmonize the stages of pretreatment and desalination and ensure reagent-free and ecological cleanliness of the entire technological process. In our opinion, the development of methods for adjusting the pH of natural waters and technological solutions before their desalination and deionization is more in demand.

## Data Availability

Not applicable.
